# Molecular identification of differentially regulated genes in the hydrothermal-vent species *Bathymodiolus thermophilus *and *Paralvinella pandorae *in response to temperature

**DOI:** 10.1186/1471-2164-10-222

**Published:** 2009-05-13

**Authors:** Isabelle Boutet, Didier Jollivet, Bruce Shillito, Dario Moraga, Arnaud Tanguy

**Affiliations:** 1 CNRS, UMR 7144, Adaptation et Diversité en Milieu Marin, Station Biologique, de Roscoff, 29682 Roscoff, France; 2 UPMC Univ Paris 06, UMR 7144, Equipe Génétique et Adaptation en Milieu Extrême, Station Biologique de Roscoff, 29682 Roscoff, France; 3 UPMC Université Paris 6, UMR 7138, Systématique, Adaptation et Evolution, 75005 Paris, France; 4 UMR CNRS 6539, Laboratoire des Sciences de l'Environnement Marin, Institut Universitaire Européen de la Mer, Université de Bretagne Occidentale, Place Nicolas Copernic, 29280 Plouzané, France

## Abstract

**Background:**

Hydrothermal vents and cold seeps represent oases of life in the deep-sea environment, but are also characterized by challenging physical and chemical conditions. The effect of temperature fluctuations on vent organisms in their habitat has not been well explored, in particular at a molecular level, most gene expression studies being conducted on coastal marine species. In order to better understand the response of hydrothermal organisms to different temperature regimes, differentially expressed genes (obtained by a subtractive suppression hybridization approach) were identified in the mussel *Bathymodiolus thermophilus *and the annelid *Paralvinella pandorae irlandei *to characterize the physiological processes involved when animals are subjected to long term exposure (2 days) at two contrasting temperatures (10° versus 20°C), while maintained at *in situ *pressures. To avoid a potential effect of pressure, the experimental animals were initially thermally acclimated for 24 hours in a pressurized vessel.

**Results:**

For each species, we produced two subtractive cDNA libraries (forward and reverse) from sets of deep-sea mussels and annelids exposed together to a thermal challenge under pressure. RNA extracted from the gills, adductor muscle, mantle and foot tissue were used for *B. thermophilus*. For the annelid model, whole animals (small individuals) were used. For each of the four libraries, we sequenced 200 clones, resulting in 78 and 83 unique sequences in mussels and annelids (about 20% of the sequencing effort), respectively, with only half of them corresponding to known genes. Real-time PCR was used to validate differentially expressed genes identified in the corresponding libraries. Strong expression variations have been observed for some specific genes such as the intracellular hemoglobin, the nidogen protein, and Rab7 in *P. pandorae*, and the SPARC protein, cyclophilin, foot protein and adhesive plaque protein in *B. thermophilus*.

**Conclusion:**

Our results indicate that mussels and worms are not responding in the same way to temperature variations. While the results obtained for the mussel *B. thermophilus *seem to indicate a metabolic depression (strong decrease in the level of mRNA expression of numerous genes) when temperature increased, the annelid *P. pandorae *mainly displayed a strong regulation of the mRNA encoding subunits and linkers of respiratory pigments and some proteins involved in membrane structure. In both cases, these regulations seem to be partly due to a possible cellular oxidative stress induced by the simulated thermal environment (10°C to 20°C). This work will serve as a starting point for studying the transcriptomic response of hydrothermal mussels and annelids in future experiments in response to thermal stress at various conditions of duration and temperature challenge.

## Background

The distribution of terrestrial, as well as marine, organisms is strongly influenced by environmental factors (variation, gradient, intensity), and temperature was identified as one of the most important. Temperature is known to affect the spatial distribution of species according to their thermal tolerance [1]. Environmental temperature challenge has a direct impact on ectothermic marine animals, and influences biological functions at all levels, from molecules to whole organisms [2,3]. Variation and/or gradients of temperature are considered as strong selective factors [4,5]. Numerous studies dealing with the effect of temperature have been conducted on coastal marine species at the molecular, physiological and protein levels [6,7]. An elevation of temperature can increase reaction rates and affect reaction equilibrium through higher kinetic energy. In addition, high temperatures induce protein denaturation, resulting in complete and often irreversible loss of function [2].

Hydrothermal vents and cold seeps represent oases of life in the deep-sea environment but are also characterized by challenging environmental conditions, when compared to the surrounding deep-sea. The hydrothermal fluid is the result of chemical modifications of the deep-sea water by interaction with the hot rocks (near the magma chamber) during a long percolating period through the oceanic crust. The resulting fluid is a hot water (up to 400°C), often anoxic, acidic (pH 2), and containing high concentrations of methane, carbon dioxide, sulfide, heavy metals, and arsenic-containing compounds [8-10]. As it comes out of the sea-floor, the hydrothermal fluid is chaotically mixed with the cold, more oxygenated deep-sea water. Abiotic factors such as changes in fluid flow, temperature and chemical composition affect species distributions at vents [11-13]. The endemic hydrothermal vent organisms live in the mixing zone according to their tolerance to – and requirements for- various factors (temperature, pH, O_2 _concentration...). However, the effect of temperature variations on hydrothermal vent organisms has not been well studied, particularly at the molecular level. Available data on the vent mussel of genus *Bathymodiolus *are mainly focused on the effect of heavy metals and oxidative stress on enzymatic activities [14] and specific gene expression (e.g. metallothioneins) [15]. A few immune system-relevant genes have also been described [16], and a cDNA library from *Bathymodiolus azoricus *has been partially sequenced [17]. Numerous studies also described these mussels' symbiosis with chemoautotrophic bacteria, often at both the phylogenetic and the biochemical levels [18-20]. HSP70, a typical thermal stress marker, has also been studied in hydrothermal vent species in response to temperature and other parameters. The HSP70 gene has been characterized in the vent shrimps *Mirocaris fortunata *and *Rimicaris exoculata *[21], in the vent annelid *Paralvinella grasslei *[22], and variations in HSP70 protein content were correlated to variations of environmental parameters for the vent mussel *B. azoricus *[23]. In Alvinellidae, a polychaete family endemic of hydrothermal vents that includes the genera *Alvinella *and *Paralvinella*, hemoglobins were largely studied at both the molecular and the functional levels [24]. Recent studies demonstrated the temperature preference of vent worm species by incubating them along a thermal gradient ranging from 15 to 60°C [25,26]. The existence of thermally sensitive alleles was shown for some proteins in *P. pandorae *compared to other species living at higher temperatures, such as *A. pompejana*, *P. sulfincola *or *P. hessleri *[27]. These data could be partly related to the species distribution in their respective habitats that mostly differ by their temperature range.

The vent mussel *B. thermophilus *is found under a wide range of environmental conditions on the East Pacific Rise. It may occur with tubeworms in areas of active hydrothermal flow where temperature and hydrogen sulfide concentrations are high, as well as in areas of diffuse flow, where the temperature and the concentration of hydrogen sulfide are much lower [28]. Studies have shown that *B. thermophilus *can quickly adapt to a wide range of environmental conditions (especially chemical changes) but usually lives between 4 and 14°C [29]. No studies report a thorough characterization of *P. pandorae irlandei*'s habitat but this species is mainly found in the cracks at the base of the tubes of the vestimentiferan tubeworm *Tevnia jerichonana *[30] where it encounters temperatures ranging from 5 to 22°C [31]. Because temperature highly fluctuates in time, a thermal regime of 20°C therefore represents an upper thermal condition for both species whereas an average temperature of 10°C could be more related to their thermal preference. In this work, we used a suppression subtractive hybridization (SSH) approach to (1) verify if such a temperature challenge induces a transcriptomic response, (2) characterize genes and the physiological processes involved in response to temperature variations, and (3) compare common and specific responses attributable to these two highly-divergent species.

## Results

### Mussel SSH libraries sequencing results

The sequencing of 200 clones from the forward (individuals incubated at 10°C versus individuals exposed at 20°C) SSH library, as well as from the reverse library (individuals incubated at 20°C versus individuals exposed at 10°C), allowed the identification of 78 unique sequences for *B. thermophilus *(19% of the whole sequencing effort: other sequences are redundant). Table 1 shows the unique sequences obtained in both SSH libraries with the best E-values for the sequences when identified after Blast analysis. The identified sequences indicate that temperature regulates genes involved in various cell functions: (1) cell cycle regulation, DNA repair, protein regulation and transcription (8.5%), (2) mitochondrial respiratory chain (4%), (3) metabolism (6.4%), (4) stress response and detoxification (0.5%), (5) cell communication, membrane receptors and immune system (10.2%), (6) cytoskeleton production and maintenance (7.5%), (7) ribosomal proteins (10%), (8) proteins of unknown function (0.5%) and (9) unknown sequences (52%). A higher proportion (60%) of the genes identified in our libraries were expressed in mussel exposed to 10°C and were mainly involved in metabolism, stress response, transcription and cytoskeleton.

**Table 1:** Regulated genes identified in the SSH libraries of thermally challenged *Bathymodiolus thermophilus *with significant database matches (only sequences with E-value above 0.005 are shown).

**Homolog (protein); Blastx value**	**Homolog species**	**Insert size (bp)**	**Accession number**	**SSH library**
**Cell cycle, DNA repair, protein regulation and transcription**				

Elongation factor 1 alpha; 1e-14	*Mytilus galloprovincialis*	428	GH196568	forward

Elongation factor beta; 2e-05	*Strongylocentrotus purpuratus*	153	GH196577	forward

Elongation factor-2; 9e-89	*Hutchinsoniella macracantha*	577	GH196570	forward

S-cyclophilin; 2e-14	*Gallus gallus*	384	GH196575	forward

H3 histone; 4e-43	*Mus musculus*	538	GH196576	reverse

Myc homolog; 7e-10	*Crassostrea virginica*	704	GH196563	reverse

**Respiratory chain**				

Cytochrome c oxidase subunit II; 5e-32	*Lampsilis ornata*	424	GH196573	reverse

Cytochrome c oxidase subunit III; 1e-20	*Arbacia lixula*	260	GH196584	reverse

ATP synthase; 3e-21	*Bos taurus*	415	GH196588	reverse

**Metabolism**				

S-adenosylhomocysteine hydrolase 2e-60	*Branchiostoma belcheri tsingtaunese*	505	GH196574	forward

Cytosolic Lactate/malate dehydrogenase; 2e-14	*Caenorhabditis elegans*	421	GH196590	forward

Arginine kinase;	*Octopus vulgaris*	135	GH196579	forward

Anhydrase carbonic 2; 2e-02	*Oncorhynchus mykiss*	114	GH196567	forward

Delta-5-desaturase; 4e-25	*Strongylocentrotus purpuratus*	195	GH196582	forward

**Stress response and detoxication**				

Glutathione peroxidase; 0.001	*Bombyx mori*	101	GH196562	forward

Heat Shock Protein 90; 4e-21	*Tetraodon nigroviridis*	156	GH196561	forward

**Cell communication, membrane receptors, immune system**				

Kalicludine 1; 3e-16	*Anemonia sulcata*	406	GH196560	reverse

Techylectin-5A; 8e-29	*Tachypleus tridentatus*	337	GH196558	reverse

Electron-transfer-flavoprotein; 4e-57	*Strongylocentrotus purpuratus*	535	GH196557	reverse

Itm1 protein, 4e-27	*Xenopus laevis*	179	GH196556	reverse

C1q-like adipose specific protein; 2e-07	*Salvelinus fontinalis*	570	GH196594	reverse

β-1,3-N-acetylglucosaminyltransferase 6; 5e-48	*Homo sapiens*	586	GH196571	reverse

Secreted protein, acidic, rich in cysteine SPARC; 2e-20	*Artemia franciscana*	523	GH196580	forward

Defensin; 1e-04	*Haliotis discus hannai*	105	GH196559	forward

**Cytoskeleton production and maintenance**				

α-2-tubulin; 4e-27	*Gecarcinus lateralis*	348	GH196585	forward

Actin; 1e-35	*Mytilus galloprovincialis*	541	GH196578	forward

Adhesive plaque matrix protein; 6e-06	*Mytilus galloprovincialis*	291	GH196589	forward

Foot protein 2; 1e-6	*Mytilus edulis*	108	GH196581	forward

Pedal retractor muscle myosin; 6e-50	*Mytilus galloprovincialis*	426	GH196564	forward

Hemicentin; fibulin 6; 2e-14	*Rattus norvegicus*	304	GH196587	reverse

**Ribosomal proteins**				

Ribosomal protein L3; 2e-55	*Argopecten irradians*	495	GH196591	forward

Ribosomal protein L4; 4e-80	*Latimeria chalumnae*	926	GH196586	forward

Ribosomal protein S14; 3e-04	*Rattus norvegicus*	256	GH196555	forward

Ribosomal protein S15; 1e-64	*Argopecten irradians*	465	GH196566	forward

Ribosomal protein S19; 2e-09	*Chlamys farreri*	362	GH196569	forward

Ribosomal protein S25; 1e-28	*Crassostrea gigas*	379	GH196583	forward

Ribosomal protein L15; 7e-16	*Siniperca kneri*	214	GH196565	reverse

QM protein; 7e-11	*Apis mellifera*	622	GH196572	reverse

**Unknown function**				

CG33171-PC, isoform C; 4e-07	*Drosophila melanogaster*	615	GH196592	reverse

Repeat organellar protein-related; 1e-05	*Plasmodium yoelii yoelii*	765	GH196595	reverse

**Unidentified sequences**				

39 sequences			GH196596 to GH196632	

Forward: genes over-expressed at 10°C versus 20°C;

Reverse: genes over-expressed at 20°C versus 10°C.

### Annelid SSH libraries sequencing results

The sequencing of 200 clones from the forward (individuals incubated at 10°C versus individuals exposed at 20°C) SSH library, as well as from the reverse library (individuals incubated at 20°C versus individuals exposed at 10°C) allowed the identification of 83 different gene sequences for *P. pandorae *(20% of the whole sequencing effort: the other sequences are redundant). Table 2 shows the unique sequences obtained in both SSH libraries with the best E-values for the sequences when identified after Blast analysis. The identified sequences indicate that temperature regulates the expression of genes involved in various cell functions: (1) respiratory chain (9.6%), (2) sulfur and oxygen transport (8.4%), (3) metabolism (2.4%), (4) cell communication, membrane receptors and immune system (4.8%), (5) cytoskeleton production and maintenance (3.6%), (6) ribosomal proteins (8.4%), (7) proteins of unknown function (2.4%) and (8) unknown sequences (60.4%). Interestingly, most transcripts identified in the reverse subtractive library correspond to genes involved in the respiratory chain and in oxygen transport. A high proportion of unknown sequences were also obtained limiting the identification of new temperature-regulated putative candidate gene.

**Table 2:** Regulated genes identified in the SSH libraries of thermal exposed *Paralvinella pandorae irlandei *with significant database matches (E-value above 0.005).

**Homolog (protein); Blastx value**	**Homolog Species**	**Insert size (bp)**	**Accession number**	**SSH library**
**Respiratory chain**				

Cytochrome c oxidase subunit I; 1e-73	*Littorina saxatilis*	772	GH196478	reverse

Cytochrome oxidase subunit II; 1e-46	*Lumbricus terrestris*	715	GH196499	reverse

Cytochrome c oxidase subunit III; 2e-34	*Albula glossodonta*	457	GH196480	reverse

Cytochrome c oxidase polypeptide Va; 1e-20	*Strongylocentrotus purpuratus*	149	GH196490	reverse

Ubiquinol-cytochrome c reductase complex; 4e-23	*Mus musculus*	537	GH196502	reverse

Cytochrome b; 1e-23	*Urechis caupo*	258	GH196495	reverse

NADH dehydrogenase subunit 1; 4e-48	*Urechis caupo*	571	GH196493	reverse

NADH dehydrogenase subunit 6; 7e-05	*Clymenella torquata*	190	GH196501	reverse

**Sulfur and oxygen transport**				

Hemoglobin A2c chain; 3e-31	*Arenicola marina*	569	GH196487	reverse

Hemoglobin B1 chain precursor; 4e-21	*Arenicola marina*	326	GH196479	reverse

Hemoglobin B2 chain; 1e-12	*Arenicola marina*	516	GH196491	reverse

Hemoglobin linker LY precursor; 5e-86	*Riftia pachyptila*	529	GH196505	reverse

Extracellular globin linker L1 precursor; 0.002	*Alvinella pompejana*	335	GH196504	reverse

Hemoglobin linker L2 precursor; 0.005	*Alvinella pompejana*	727	GH196483	forward

Intracellular hemoglobin; 5e-41	*Alvinella pompejana*	547	GH196492	forward

**Metabolism**				

Trypsin; 6e-60	*Litopenaeus vannamei*	661	GH196494	reverse

S-adenosylhomocysteine hydrolase; 2e-66	*Branchiostoma v*	***529***	GH196476	forward

**Cell communication, membrane receptors, immune system**				

Lipid binding protein 9; 5e-05	*Caenorhabditis elegans*	463	GH196481	forward

GTP-binding protein (rab7); 7e-89	*Canis familiaris*	641	GH196489	forward

Xylan endohydrolase isoenzyme, 6e-22	*Arabidopsis thaliana*	455	GH196472	reverse

Cyclophilin B; 4e-14	*Gallus gallus*	384	GH196477	reverse

**Cytoskeleton production and maintenance**				

Chymotrysin; 4e-11	*Lumbricus rubellus*	230	GH196484	forward

Secreted nidogen domain protein; 4e-08	*Strongylocentrotus purpuratus*	325	GH196473	forward

Actin A1; 2e-35	*Haliotis iris*	541	GH196475	forward

**Ribosomal proteins**				

Ribosomal protein L5; 3e-58	*Rattus norvegicus*	423	GH196497	reverse

Ribosomal protein L28; 6e-07	*Haliotis asinina*	262	GH196486	reverse

Ribosomal protein L24; 1e-38	*Danio rerio*	439	GH196482	forward

Ribosomal protein S3; 3e-30	*Crassostrea gigas*	205	GH196474	forward

Ribosomal protein S16; 2e-41	*Gallus gallus*	337	GH196500	forward

Ribosomal protein SA; 8e-43	*Xenopus tropicalis*	514	GH196496	forward

Ribosomal protein P1; 2e-11	*Drosophila yakuba*	444	GH196498	forward

**Unknown function**				

CG14235-PA, isoform A; 1e-27	*Tribolium castaneum*	483	GH196488	forward

CBG19860; 2e-08	*Caenorhabditis briggsae*	266	GH196485	reverse

**Unidentified sequences**				

50 sequences			GH196507 to GH196554	

Forward: genes over-expressed at 10°C versus 20°C; Reverse: genes over- expressed at 20°C versus 10°C.

### Relative expression level of some target genes

Levels of expression of 20 transcripts obtained in the *B. thermophilus *library and 14 transcripts identified in the *P. pandorae *library were quantified from pools of individuals of each thermal condition by using real-time PCR. For all the genes, we showed that the differential expression observed for the genes corresponds to the library they came from. In addition, stronger differential expressions were observed in *B. thermophilus *for metabolic genes such as S-adenosylhomocysteinase-hydrolase, arginine kinase or Δ5-desaturase, and some genes such as secreted protein acidic rich in cystein (SPARC, a basal membrane component), elongation factor beta, actin and proteins related to mussel mobility (foot protein and adhesive plaque matrix protein) (Table 3). While it was identified in the reverse library, the gene encoding the kalicludine did not display a clear differential expression between the two experimental conditions. In *P. pandorae*, differences between levels of expression of the two sets of experimented individuals were higher than those observed in mussels. Some genes also showed a very high level of expression in samples exposed to 10°C as compared to those exposed at 20°C, especially for the secreted nidogen domain protein, Rab7, the intracellular hemoglobin and the ribosomal protein S16 that were strongly inhibited in annelids exposed to 20°C. Almost all genes encoding subunits of hemoglobins (except linker L2) displayed a coherent expression pattern of up-regulation in annelids exposed to 20°C (Table 4).

**Table 3:** mRNA expression of genes in *B. thermophilus *presented as a fold-change value with animals exposed at 10°C as a calibrator.

**Genes**	**Fold-change **(calibration to 10°C)
***Down-regulated at 20°C***	
Defensin	1192.69
Elongation Factor beta	4.59
Ribosomal protein L3	6.23
Foot protein	3.36
Pedal retractor muscle myosin	5.17
Adhesive plaque matrix protein	2.83
Actin	4.03
Secreted Protein, Acidic, Rich in Cystein (SPARC)	7.21
S-adenosylhomocysteine hydrolase	5.24
Cyclofilin S	4.35
Δ5-desaturase	8.28
Carbonic anhydrase 2	11.39
Adenylate kinase	6.59
Gluthatione peroxidase	6.28
Cytosolic malate dehydrogenase	8.75
HSP90	6.23
BthermEST1	2.57

***Up-regulated at 20°C***	
Kalicludine	1.06
Myc homolog	12.30
Techylectin 5A	6.63

**Table 4:** mRNA expression of genes in *P. pandorae *presented as a fold-change value with animals exposed at 10°C as a calibrator.

**Genes**	**Fold-change **(calibration to 10°C)
***Up-regulated at 20°C***	
Hemoglobin A2c	250
Hemoglobin B2	31.25
Linker L1	142.86
PpandEST2	13.33
Xylan endohydrolase	5.13

***Down-regulated at 20°C***	
Intracellular Hemoglobin	1226.22
Linker L2	4.03
Secreted Nidogen domain protein	1278.29
S-adenosylhomocysteine hydrolase	35.51
Chymotrypsinogen	20.46
PpandEST1	1.53
Ribosomal protein S16	1640.59
Lipid binding protein	1.80
Rab 7	2469.49

## Discussion

In the present paper, we described and analyzed gene expression in two hydrothermal species in response to two temperatures, one included in the range of temperature encountered by both species (10°C), and the other near (for worms) or above (for mussels) their thermal limit range (20°C). The following parts of the discussion deal with the respective responses of these two species in terms of differences and common features typifying their ability to adapt different thermal regimes.

### Unidentified sequences

Over 50% of the sequences for each species could not be identified based on homologies. This may be due to the limited amount of data available for invertebrates, or to the SSH protocol itself that requires the use of a restriction enzyme, possibly leaving only the UTRs for cloning. This may explain that we did not obtain typical heat stress proteins such as the inducible heat shock protein 70 (HSP70). This could also be due to the long term acclimation (about 2 days) offered to the animals leading to an attenuation of the stress machinery with time. None of the SSH libraries (mussel and annelid) indeed contained mRNA coding for any HSP70s, that are involved in the protection of other proteins from denaturation caused by a variety of stressors [32,33]. Available data dealing with HSP70 in hydrothermal species were only obtained from polychaetes, mussels and shrimps exposed to brief heat shocks [21,22,34]. In previous studies, a positive correlation between the levels of DNA strand breakage and HSP70 expression in response to decompression stress were also found by Pruski and Dixon [35]. In the shrimp *Rimicaris exoculata*, regulation of HSP70 in response to temperature was detected at the protein level [21], but no data related to HSP70 gene regulation is available so far.

### Is *P. pandorae *better adapted to higher temperature than *B. thermophilus*?

Metabolic adjustments in response to thermal challenges are essential for aquatic ectotherms, whose body temperature fluctuates over the full range of temperature in their habitat [36]. Temperature can also influence metabolic regulation, eliciting transition to anaerobiosis even in oxygenated waters [37]. A high thermal sensitivity of metabolism over the environmental range is associated to increased long-term metabolic costs and with a lower tolerance to extreme temperatures. Classically, lowering metabolic rate and, thus energy saving is also considered as one of the most important adaptations for hypoxia endurance [38,39]. Adaptation to these conditions has resulted in reduced growth rates, as well as reduced development and metabolism [40]. Previous studies conducted on the marine gastropod *Littorina saxatilis *showed that an acute long-term temperature increase could disturb metabolism, leading to progressive metabolic depression and adverse changes in the cellular energy status due to its transition to partial anaerobiosis [41]. Antarctic marine species are also much less capable to survive elevated temperatures [42] and calculated temperature envelopes for these organisms were 2–4 times smaller than those for temperate species [43]. Studies on the Mediterranean mussel *Mytilus galloprovincialis *showed that a long acclimation of up to 30 days at high temperatures (18 to 30°C) leads to behavioral (increase of duration of valve closure), metabolic (metabolic depression with a shift from aerobic to anaerobic metabolism) and molecular (increase in HSPs protein levels) responses [44]. Interestingly, the deep-sea mussel *B. thermophilus *seems to share some general features with organisms living in polar oceans that are characterized by very stable low temperatures, below 5°C. We first hypothesized that annelids were able to cope with a larger range of temperature compared to the mussels, because they were described as early colonizers of new chimneys at hydrothermal vents [45], and thus able to sustain highest temperatures, at least over short periods of time. While few genes seem to be regulated similarly between the two species (actin, mitochondrial cytochrome oxydase, S-adenosylhomocysteine hydrolase), most cell functions are very dissimilar, suggesting a different response of these organisms to temperature. The genes that were identified in the mussel libraries could indicate that this species tends to react as a stenoecious species rather than an euryecious species (as could be expected for organisms living in a highly fluctuating environment). A general depression is indeed observed in expression of *Bathymodiolus *genes involved in transcription/translation, mobility, energetic metabolism, and oxidative stress in response to temperature increase. Conversely, genes involved in cell disorder and immune system (ie myc-homolog, kalicludin...) are up-regulated at the highest temperature. No similar pattern is observed in *P. pandorae*, leading to the hypothesis that this species could better adapt to high temperatures.

A long exposure at a temperature of 20°C (43 hours) clearly appears to be a thermal physiological limit for *B. thermophilus *that lives in colder habitats. As mussels encounter short pulses of hot water under *in situ *conditions, it seems that the duration of the heat exposure is critical and probably more important than the temperature value itself. According to the experimental conditions used for this study, it remains difficult to evaluate how long *B. thermophilus *is really able to withstand a temperature of 20°C without severe physiological damage. Complementary experiments such as the determination of differential mortality kinetic in longer-term exposures and at different ranges of temperatures have to be performed in further studies to better understand the thermal resistance/response/adaptation of this species. As all individuals survived for about 2 days (43-hour experiment), *B. thermophilus *can deal with thermal stress for at least few days but it is not clear whether they can then recover from such stress.

The hypothesized limited adaptation of *B. thermophilus *to high temperature is also supported by a decrease of expression of nearly all genes identified in the mussels exposed to 20°C, a pattern indicative of global metabolic depression. Among these genes, there were many ribosomal proteins and some elongation factors, indicating that the protein synthesis pathway was clearly involved in response to temperature. However, different ribosomal proteins are identified in both reverse and forward libraries showing a complex regulatory process (or the absence of regulation) in intra-molecular interactions in ribosomes. This result is commonly observed in transcriptomic studies in mollusks in response to various environmental parameters [46-49]. A higher number of ribosomal proteins were however identified in the mussel forward libraries suggesting a possible metabolic depression in samples exposed to 20°C when compared to those exposed at 10°C. In *P. pandorae *libraries, similar pattern of down regulation at 20°C of 5 ribosomal proteins among the 7 identified is observed.

More specifically, it is noteworthy that several genes of the mussel energetic pathways were down-regulated. Among them, arginine kinase (ArgK) and cytosolic malate dehydrogenase (cMDH) are found to be down regulated in mussels exposed to 20°C. ArgK catalyzes the transfer of phosphate between ATP and arginine (arginine phosphate + MgADP^- ^+ H^+ ^↔ arginine + MgATP^2-^), and plays a critical role in cellular energy metabolism in invertebrates [50]. It also serves as an energy reserve because it can readily transfer phosphor-arginine to ATP when energy is needed [51,52]. However, it was never found associated with thermal stress. To our knowledge, no data on the thermal regulation of mRNA expression of ArgK has been reported to date. Its regulation has mostly been studied at a protein level and this is the only phosphagen kinase known in crustaceans and mollusks. ArgK is indeed regulated in crustaceans and mollusks under hypoxia [53,54]. In the crustacean *Marsupenaeus japonicus*, the up-regulation of ArgK under hypoxia may represent a provision for oxygen recovery after a short period of hypoxia [54]. The second metabolic enzyme is the cytosolic malate dehydrogenase, which catalyzes the dehydrogenation of malate (malate + NADP^+ ^↔ oxaloacetate + NADPH + H^+^). It plays a major role in a number of metabolic pathways, including the malate-aspartate (or NADH) shuttle and the acetate shuttle active in lipogenesis, amino acid synthesis and gluconeogenesis. cMDH is an interesting candidate gene to study adaptation to temperature as this enzyme showed differences in the effects of temperature on kinetic properties in shallow water species [7,55,56]. Even though no cMDH mRNA expression has been reported in these studies, differences in protein properties strongly suggest a clear involvement of this key gene in response to temperature. The down-regulation of both ArgK and cMDH in *B. thermophilus *could lead to a decrease of mitochondrial respiration, leading to a lower ATP production, and resulting in the establishment of a global metabolic depression in response to temperature.

The cDNA coding for a HSP90 was found in the mussel SSH libraries. However, HSP90 displayed a down-regulation at 20°C when compared to 10°C suggesting that the process of protein re-naturation was probably over. HSP90 proteins have key roles in signal transduction, protein folding, protein degradation, and morphological evolution [57-59]. HSP90 is up-regulated in response to heat stress in *Drosophila subobscura *[60], the whitefly *Bemisia argentifoli *[61], and the flesh fly, *Sarcophaga crassipalpis *[62]. It is induced by thermal stress in the Goby fish but could also decrease in expression to a normal level during the acclimatization process [63]. In *M. galloprovincialis*, both HSP70 and HSP90 protein expression were shown to increase in response to long-term thermal challenge [44].

### Does temperature generate a stronger oxidative stress in mussels than in annelids?

We identified several genes that are classically expressed in response to oxidative stress in the mussel libraries but not in the annelid libraries, suggesting a differential behavior of both species. Two main hypotheses can explain the presence of an oxidative stress: (1) a direct effect of temperature changes on lipid composition or (2) variations of the oxygen concentration during experiments. In the first hypothesis, temperature directly affects cells by modifying membrane composition through replacement of unsaturated fatty acids at low temperatures towards saturated fatty acids at high temperatures [64], and secondly by inducing apoptosis *via *activation of the sphingomyelin pathway that leads to the process of lipid peroxidation [65]. Many biological structures, such as enzymes and lipid bilayer membranes, depend on a particular degree of molecular instability or fluidity, which is directly affected by temperature. In the particular case of our experimented hydrothermal species, lipids of cell membrane bilayers must be both fluid and structurally coherent to form a functional membrane, a characteristic very sensitive to temperature change [2]. Lipid peroxyl radicals (LPO) are the result of a reaction between lipid and oxygen and are known to damage cells by changing the fluidity and permeability of the membrane and/or by directly damaging DNA and other intracellular molecules, such as proteins [66]. As a consequence of the lipid peroxidation process, superoxide anion radicals can be produced. Lipid peroxidation has been studied in hydrothermal vent mussels and high levels of LPO were detected in *B. azoricus *in response to a strong effect of environmental heavy metal concentrations [67]. Recently, heavy metal stresses, such as copper exposure, or changes in hydrostatic pressure were also shown to produce LPO in *B. azoricus *[68]. Fatty acid desaturases are very important during the process of fatty acid metabolism that contributes to the structural and functional maintenance of biological membranes in living organisms. The down-regulation of Δ5-desaturase mRNA expression of *B. thermophilus *exposed to 20°C is consistent with a modification of membrane lipid content. We also identified a gene encoding SPARC, which is more expressed in mussels incubated at 10°C when compared to those exposed to 20°C. SPARC is classically known to modulate cellular interaction with the extracellular matrix through interactions with proteins such as laminins and collagen [69,70]. SPARC was also shown to be up-regulated in response to heat-shock and other stresses [71,72]. SPARC also possesses a chaperone-like activity *in vitro *suggesting its involvement in stress response [73]. Its down-regulation at 20°C is coherent with results observed by previous authors and could reflect a strong disorder in membrane composition due to the high temperature.

In the second hypothesis, the generation of reactive oxygen species (ROS) as side products of electron transfer during aerobic metabolism [74] can explain the regulation of genes encoding protective proteins in mussels. Here, oxidative stress can be due to the experimental conditions used where sea-water was at a low-oxygen concentration (below 120 μM) associated with a consumption by animals and the effect of temperature. In the presence of low oxygen concentration or anoxic conditions, organisms use anaerobic metabolism and annelids and mollusks are able to use more efficient mitochondrial pathways of fermentation [75-77]. Under normal physiological conditions, anaerobic metabolism produces free radicals, and cells tend to maintain a balance between generation and neutralization of ROS. When organisms are subjected to xenobiotics, temperature increase or anoxia events, the generation of ROS can exceed the scavenging capacity [78]. All organisms possess their own cellular antioxidant defense system, composed of both enzymatic (superoxide dismutase, catalase and glutathione peroxidases) and non-enzymatic (glutathione, vitamins...) components. Glutathione peroxidases (GPx), that have protective roles against oxidative stress, have been identified in *B. thermophilus *libraries suggesting an oxidative stress as a direct or indirect result of temperature challenge. Surprisingly, GPx expression is lower in mussels exposed to 20°C than those exposed to 10°C despite the fact that oxidative stress is supposed to be stronger at 20°C, supporting the idea that *B. thermophilus *is no longer able to regulate expression of oxidative stress related genes. We also identified a gene encoding a myc homolog which is up-regulated at 20°C compared to 10°C-exposed mussels. This protein belongs to a transcription factor family and is involved in the cell division control. Myc and its binding partners regulate the expression of a large number of genes that regulate diverse functions, including protein synthesis, apoptosis, and DNA and energy metabolism [79-81]. Generally speaking, over-expression of a myc-homolog enhances apoptosis by acting as a transcription repressor [82,83]. In bivalves, c-myc has previously been shown to be up-regulated by hypoxia [49] and hydrocarbon stresses [46]. Identification of c-myc in mussel exposed to 20°C seems to be indicative of the very poor biological condition of the 20°C-exposed individuals and thus in accordance with the hypothesis of a low tolerance of *B. thermophilus *to extended exposure to high temperature.

A gene that is involved in adenosine metabolism, and that has previously been shown to be regulated in response to hypoxia, has also been found in both mussel and annelid libraries. This enzyme called *S*-adenosylhomocysteinase hydrolase (SAHH, EC 3.3.1.1) catalyses the reversible hydrolysis of *S*-adenosylhomocysteine to form homocysteine and adenosine [84]. AdenosineMethionine/AdenosineHomocysteine turnover is believed to play a critical role in methionine metabolism and the regulation of biological methylation processes. Tissue hypoxia induces a variety of functional changes, including enhanced transcriptional activity associated with high transmethylation activity (e.g. mRNA cap methylation) in the nucleus. Disturbance in DNA methylation pattern has previously been observed in response to various stressors, such as heavy metals, as a consequence of toxicity [85,86]. In both our species, the mRNA expression of this gene is lower in animals exposed to 20°C than to 10°C. This regulation of SAHH mRNA expression supports the hypothesis of a response to a direct or indirect oxidative stress. Presence of SAHH in response to temperature also illustrates the importance of methylation processes as a response to temperature increase. Generally speaking, a strong DNA methylation leads to a decrease or an inactivation of gene expression. Methylation processes regulation could be an interesting type of response to temperature in hydrothermal species.

### Specific responses to temperature in mussels and annelids

In the mussel libraries, we interestingly identified three down-regulated genes at 20°C that are involved in foot activity (foot protein and pedal retractor muscle myosin) and byssus activity (adhesive plaque matrix protein). These results are in sharp contrast with previous studies performed on the brackish-water mussel, *Mytilopsis leucophaeata *that showed an increase in foot activity index and byssus thread production in response to thermal challenge [87]. Authors demonstrated that both foot activity and byssus production were higher when temperature increased from 4 to 20°C, remained stable between 20 and 28°C, and then strongly decreased beyond 28°C. This species commonly lives in a range of temperatures comprised between 4°C (winter) and 20°C (summer). Mussels of the genus *Bathymodiolus *are able to change location when the conditions are not adequate [88]. This can be viewed as an escape response in the presence of stress factors. They also probably use this mobility to optimize their position in the hydrothermal fluid in order to acquire the sulfide (and/or methane for some species) they need to feed their symbionts. The decrease of the expression of genes encoding proteins related to mobility in mussels exposed at 20°C, again reflects the poor physiological condition of these individuals, since mussels usually live in colder waters (4 to 14°C).

In the annelid SSH libraries, we identified several genes encoding various extracellular globin chains (B1, A2 and B2), one intracellular globin and also three linkers called linker L1, linker L2 and linker LY. These results illustrate a strong involvement of respiratory pigment in general and in particular of the hexagonal bilayer hemoglobin (HBL-Hb) in response to temperature in this species. In Alvinellidae, respiratory gas transport is performed by the blood and the coelomic fluid, and three main types of globins are present: non-circulating in the cytoplasm, circulating and intracellular in the coelom, and extracellular in the vascular system [24]. Earlier work reported the temperature effect on both the function and the stability of Hbs in the annelid *Alvinella pompejana*, under atmospheric pressure and for temperatures ranging from 10°C to 40°C. These Hbs are able to maintain a capacity to reversibly bind oxygen *in vitro *over this range. At 50°C, the Hbs are oxidized and aggregated during the de-oxygenation and the re-oxygenation [89]. These results are in agreement with the hypothesis that annelids, even if they are able to withstand a strong thermal stress [25], are nonetheless unable to sustain high temperature for a long time [24]. Because *P. pandorae *lives in a relatively cold environment compared to other *Paralvinella *species, such as *P. sulfincola*, and probably do not experience very high temperatures, the involvement of Hbs in temperature response could be the result of several processes and not only driven by Hb thermostability properties. We observed a strong increase of mRNA encoding extracellular Hb subunits in individuals exposed to 20°C and conversely, a decrease of intracellular subunit mRNA expression. It has been suggested that extracellular Hbs were preferentially involved in oxygen uptake and transport, while intracellular Hbs acted as an oxygen reserve for the worm and potentially returned oxygen to the extracellular Hb [90]. Because oxygen availability decreases with temperature, *Paralvinella *increased their extracellular Hb production to optimize the oxygen uptake and transport. At the same time the intracellular Hb was down-regulated, possibly to avoid the release of O_2 _to the tissue since the worms experienced hot temperature but not hypoxia). We also observed an opposite regulation in the expression of linkers L1 and L2 in *P. pandorae *in response to thermal stress suggesting a possible rearrangement of the linker composition of the HBL-Hb molecule under temperature-induced oxidative stress. Very few studies have dealt with the linker function and regulation at a transcriptional level. In the Earthworm *Lumbricus terrestris*, linkers have been shown to exhibit a superoxide dismutase activity conferring a protection against superoxide ions for HBL-Hb molecules [91]. Linkers of the thermally-stressed alvinellids may therefore have been mobilized as an active defense against newly-produced ROS.

In the *P. pandorae *library, we also identified one gene encoding a secreted nidogen domain protein that showed a strong down-regulation at 20°C. Secreted nidogen domain protein, also known as entactin, belongs to basement membrane proteins. These membranes are made of type IV collagens and laminins, both of which exist as various isoforms in animals [92,93]. These proteins are cell-adhesive and form networks that confer mechanical stability to the basement membranes. Other ubiquitous basement membrane components are the proteoglycan perlecan and nidogen/entactin. Previous *in vitro *experiments showed that recombinant nidogen-1 interacted through different binding sites with the three main basement membrane components (laminin, collagen IV, and perlecan), and mediated the formation of ternary complexes between laminin and collagen IV [94]. These results therefore suggest that secreted nidogen domain protein is a key component of alvinellid basement membranes assembly, connecting the laminin and collagen networks, and integrating other basement membrane components as previously reported by Timpl and Brown [93]. We suggest that temperature (and/or pressure) above normal could induce strong changes in the membrane composition of the worms and therefore increase interactions between secreted nidogen domain protein, collagen and other membrane protein in order to readjust porosity/permeability. Other proteins that are thought to partially play a role in membrane component modeling have also been characterized. We identified a Ras-associated binding 7 (Rab 7) protein belonging to the Rab family. These are small GTPases of the Ras superfamily that continuously cycle between the cytosol and different membranes. The Rab family appears to be essential for the regulation of intracellular membrane traffic in mammalian cells. Rab proteins are anchored to the cytoplasmic surface of specific intracellular membrane compartments via the geranyl-geranyl group that is post-translationally added to the C-terminal cysteines and is important for their function [95]. Each Rab protein regulates one (or more) specific step of intracellular membrane traffic in eukaryotic cells, probably by assembling the general tethering/docking/fusion machinery [96]. Moreover, several lines of evidence suggest an involvement of Rab proteins in actin- and microtubule- based processes [97]. Rab7, a member of the Rab family small G proteins, has been shown to regulate intracellular vesicle traffic to late endosome/lysosome and lysosome biogenesis, but the exact roles of Rab7 are still undetermined [98,99]. Accumulating evidence suggests that each Rab protein has multiple target proteins that function in the exocytic/endocytic pathway. Because no studies showing how temperature could affect Rab 7 expression, its down regulation observed in *P. pandorae *by temperature remains difficult to explain but could be associated with results observed for nidogen protein.

## Conclusion

Our results indicate that the mussels and the worms did not cope with temperature in the same way. While the mussel *B. thermophilus *seems to show a general metabolic depression (strong decrease of mRNA expression for numerous genes), possibly due to maladaptation and cell disorders when temperature increased, the annelid *P. pandorae *mainlydisplayed a strong regulation of the mRNA encoding subunits and linkers of respiratory pigments and some proteins involved in membrane constitution. In both cases, these regulations seem to be partly due to a possible cellular oxidative stress induced by temperature increase (10°C to 20°C). The large number of unknown sequences makes definitive conclusions difficult. The data collected may contain a number of candidate genes regulated by temperature that require annotation and functional characterization before meaningful interpretation of temperature adaptation of both *P. pandorae *and *B. thermophilus *is possible. New tools, such as microarrays will help evaluate the expression and characterization of these genes. This work will serve as a starting point for studying the transcriptomic response of hydrothermal mussels and annelids in future experiments in response to thermal stress at various conditions of exposure duration and thermal level.

## Methods

### Animal sampling and treatment

Specimens of both the hydrothermal vent mussel *Bathymodiolus thermophilus *(67.8 ± 30 mm in length) and the annelid worm *Paralvinella pandorae irlandei *(≤ 10 mm in length) were collected together from the Oasis site (17°25.42S, 113°12.28W) on the East Pacific Rise during the BIOSPEEDO cruise [100] by using the telemanipulated arm of the submersible Nautile. The individuals were collected in a cold zone associated with weak diffuse flow (< 10°C). Once on board the ship, animals were immediately transferred from the insulated collection basket to the pressurized aquaria IPOCAMP™ (Incubateur Pressurisé pour l'Observation et la Culture d'Animaux Marins Profonds [101]) under an *in situ *pressure of 260 bars. Indeed, several studies showed that experiments conducted on mussels at atmospheric pressure lead to a global increase of stress parameters (lipid peroxidation, anti-oxidant enzymes activity, DNA damage) and to a limitation of stress response capacity [14,23,102]. One group of 5 mussels and 20 worms was placed at 10°C, and another group at 20°C, inside two different pressure vessels for 43 hours. The pressure vessels were operated in a flow-through mode (20 L/hour) for the first 3 hours, and then isolated. Every 15 hours, seawater was re-circulated for 3 hours, until the end of the incubation. When in flow-through configuration, the pressure aquaria were fed with sea-water aerated with a low-oxygen (8%) air mixture. Therefore, oxygen levels during the experiments were at a maximum value of about 120 μM during circulation periods, and obviously decreased when the vessels were isolated, due to oxygen consumption by experimented animals. No mortality was observed at the end of the experiments, and samples of mussels (pool of gills, foot, mantle, adductor muscle) and worms (whole organisms) were collected and immediately frozen in liquid nitrogen until use.

### RNA extraction

Total RNA was extracted from the gill, adductor muscle, mantle and foot of 5 thermally-challenged mussels, and 6 whole worms with the Trizol Reagent according to the manufacturer's instructions. Total RNAs extracted from the different tissues were pooled and poly(A+) mRNA was isolated using the PolyATtract^®^mRNA Isolation System (Promega, Madison, WI, USA) according to the manufacturer's instructions.

### Suppression subtractive hybridization

Both forward (individuals incubated at 10°C versus individuals incubated at 20°C) and reverse (individuals incubated at 20°C versus individuals incubated at 10°C) subtracted libraries were produced from 2 μg of mRNA extracted from experimented mussels and worms. First and second strand cDNA synthesis, *Rsa*I endonuclease enzyme digestion, adapter ligation, hybridization, and PCR amplification were performed as described in the PCR-select cDNA subtraction kit manual (Clontech, Palo Alto, CA, USA).

### Cloning and sequencing

The differentially expressed PCR products were ligated into a pGEM-T vector (Promega, Madison, WI, USA) and 200 white colonies per library were cultured in LB medium supplemented with 100 mg/L ampicillin. Plasmids were then extracted using an alkaline lysis plasmid minipreparation, and sequenced using the Big Dye Terminator V3.1 Kit (Perkins-Elmer) and run on an AB3100 sequencer (Applied Biosystems Perkins-Elmer).

### Sequence analysis and homology search

Chromatograms obtained after sequencing were treated with the Seqclean software (TGIR, the Institute for Genomic Research, Rockville, MD, USA) to remove vector and adaptors sequences. Cluster and contigs were then formed on each library sequence set. BLAST analyses of the sequences were performed on the NCBI server. The sequences were analyzed for homology with known sequences in databases using the BlastX and BlastN programs .

### Validation of differential expression by real-time PCR

A validation step of the differentially expressed genes identified in *B. thermophilus *and *P. pandorae *SSH libraries was carried out using real-time PCR and total RNA samples used for the SSH construction. For each sample, 5 μg of total RNA were submitted to reverse transcription using oligo-dT anchor primer (5'-GAC CAC GCG TAT CGA TGT CGA CT_(16)_V-3') and M-MLV reverse transcriptase (Promega, Madison, WI, USA). Amplification of 20 genes isolated in the SSH libraries was carried out on the cDNA from both 10 and 20°C exposed mussel samples and 14 genes were amplified on pool of cDNA from samples of annelid exposed to both 10 and 20°C. The real-time PCR assay was performed in triplicate with 4 μL cDNA (1/20 dilution) in a final volume of 10 μL using the Chromo 4™ System (BioRad). The concentrations of the reaction components were as follows: 1× ABsolute™ QPCR SYBR^® ^Green mix (ABgene, UK) and 70 nM of each primer (Tables 5 and 6). The 18S ribosomal DNA was amplified as an endogenous PCR control, under the same amplification conditions using sense (5'-AAG GGC AGG AAA AGA AAC TAA C-3') and antisense (5'-GTT TCC CTC TAA GTG GTT TCA C-3') primers. The amplification was carried out as follows: initial enzyme activation at 94°C for 15 min, then 45 cycles of 94°C for 15 sec and 60°C for 1 min. A dissociation curve was generated and PCR efficiency (E) was estimated for each primer pair by using a serial dilution of reverse transcription products. Standard curves were generated for each primer pair and E was calculated using the formula E = 10^(-1/slope)^-1. All primer pairs tested generated a single peak in the dissociation curve with a PCR efficiency estimated between 95 and 100%. Relative quantification (RQ) of each gene expression was calculated according to comparative CT method using the formula: RQ = exponential (2^-ΔΔCT^) with ΔΔCT = ΔCT(10°C) - ΔCT(20°C) and ΔCT(10°C) = CT(gene X in the 10°C sample) – CT(18S) and ΔCT(20°C) = CT(gene X in the 20°C sample) – CT(18S); 18S ribosomal DNA being used as the endogenous control.

**Table 5:** Primer sequences used in real-time PCR expression analysis for *B. thermophilus *SSH libraries validation

**Genes**	**Primer sequences**
Elongation factor beta	For: 5' GATCTTAAAAGTAAAGCTGGTCAGCAAGC 3'
	Rev: 5' AACAAATCAAAATCATCATCATCACCGCC 3'
Ribosomal protein L3	For: 5' AGATATATCGTATTGGAGAGGGATACCACACC 3'
	Rev: 5' GCTGTTGCATCCTTCTTCAATGGACCCAT 3'
Foot protein	For: 5' AATAATGGTAAATGTGTTGCTAATGGCTA 3'
	Rev: 5' CCGTATCCCCTTCTACAACATCTACCGCC 3'
Pedal retractor myosin	For: 5' AGAACCGACGAATTGGAAGAGGCCAAGAG 3'
	Rev: 5' AACAATTCAGCAGAGTAACTGCGGGCCTC 3'
Adhesive plaque matrix	For: 5' AAAAGATGTGAAGTAAACAGATGCAGCCCA 3'
	Rev: 5' CCGTATCCCCTTCTACAACATCTGCCGCC 3'
Actin	For: 5' ACGCGGGTCAGGGTCGGACGTAGCCACGC 3'
	Rev: 5' ATGGAGATCAGACGGAGATGGTCCTCCTC 3'
Adenosylhomocysteinase	For: 5' GTAAATCTTGGTTGTGCTCATGGTCATCC 3'
	Rev: 5' GATTTGAATGGTCCTTCTTTAGGTAGACC 3'
Cyclophilin S	For: 5' TTGAATAAAGCCAGATGGATGGATGGAAA 3'
	Rev:5' AAATCTTCATCGCTAGCTGCTTGTGCTTC 3'
Arginine kinase	For: 5' ATGGGTGAAGTAGCAGAATTGTGGGCTAA 3'
	Rev: 5' TCACATGCATACAATCCGACTCCGCT 3'
Glutathione peroxidase	For: 5' ATGGGCATAAACTTGGGAGACATTTT 3'
	Rev: 5' CCTAATTCTGTTGTACAAACAGGGGTATA 3'
BthermEST1	For: 5' AGTGACTTCACAACTGCCCGTATGTGGAA 3'
	Rev:5' TTGGTGCACATCATCAAGAAGGAGAGTAT 3'
Kalicludine	For: 5' CCATGCAATGAAGATTGTCTTTTGCCAAA 3'
	Rev: 5' TTTGGCAAAAGACAATCTTCATTGCATGG 3'
Defensin	For: 5' ATGTTCAAAGTAACTTTGTTCTTCGTTGG 3'
	Rev: 5' TACGATCTGCAGTGACACACGTTATG 3'
SPARC	For: 5' AACGCAGACGACCACCGTACAGACGC 3'
	Rev: 5' TATGCATCACACTTGTCTGTAATGTCAACC 3'
Δ5-desaturase	For: 5' AACGACTGGTTTACAGGGCATCTAAA 3'
	Rev: 5' TTTAGATGCCCTGTAAACCAGTCGTT 3'
Carbonic anhydrase-2	For: 5' GATGACAAGGAAGGATCTGAGCACACTCT 3'
	Rev: 5' AGAGTGTGCTCAGATCCTTCCTTGTCATC 3'
HSP90	For: 5' ATGCCTGAACCTGAAACAACTATGGATGA 3'
	Rev: 5' GAATACATCTGGGAATCTGCAGCTGGTGG 3'
Myc homolog	For: 5' TCTGTTTATGATGCCTGGGTCACTCC 3'
	Rev: 5' GGAGTGACCCAGGCATCATAAACAGA 3'
Cytosolic malate dehydrogenase	For: 5' ATGGCAGTTCCTTCAGATGGATCTTA 3'
	Rev: 5' CAATACACAAAAACAGACACTGTATACAT 3'
Techylectin 5A	For: 5' GGATATCAGGGTAATGCAGGAGATGC 3'
	Rev: 5' GCATCTCCTGCATTACCCTGATATCC 3'

**Table 6:** Primer sequences used in real-time PCR expression analysis for *P. pandorae irlandei *SSH libraries validation.

**Genes**	**Primer sequences**
Intracellular hemoglobin	For: 5' CTTGCCGATAACATTACTGCTGTTCGAGG 3'
	Rev: 5' TCGGCATCACCCGCCTTCTCCGCTACGTC 3'
Hemoglobin A2c	For: 5' GTTCTGATCATAATCGCTGTCTGTCTGG 3'
	Rev: 5' TGTAGAAGCTGGGCATTCAGCGTGTCGGG 3'
Linker L1	For: 5' ATCATGGCAGGCCTGGTGGCACTCGCCAT 3'
	Rev: 5' TCTGATCCGTCATGACAGTCGTTAGCACC 3'
Linker L2	For: 5' ATGGTTGACGATGAGATGGACTTGATGGA 3'
	Rev: 5' ATCTTATAGCTGTCTATAGTAACCCG 3'
Hemoglobin B2	For: 5' CTGGATCATCTCGGCCGTCAGCATGTTGT 3'
	Rev: 5' GTGTGGTCGAGACGCGTTGCTCGGTCCGC 3'
PpandEST 2	For: 5' CAACCATGTGCCTACTTTACCTTGTTCAG 3'
	Rev: 5' AAGAACAGCTGCTGCAGAATATCTCCCAC 3'
Xylan endohydrolase	For: 5' GAGATGGAAGAGAGAAATCCAGATTGGCT 3'
	Rev: 5' AGTTTCGCCTTCGAGTCACCGCTGTAGGC 3'
Rab 7	For: 5' CTTATTCAAGCTAGCCCACGAGATCCAGA 3'
	Rev: 5' CAACTCTCCGCTGAAGTCTTCGCCCGGTC 3'
Adenosylhomocysteinase	For: 5' ATTGTGTGCAATATTGGACATTTTGACTGTGA 3'
	Rev: 5' AGACCTAAATAGCCAGCCTGGTCATCTGA 3'
Chymotrypsin	For: 5' ACAGAGGTCGAATACGAGGTGATGACAAT 3'
	Rev: 5' ATGCCACCTGAGCTAAGAGTTCCCCATCC 3'
PpandEST 1	For: 5' GAAGCTGACCTAGCTTACGCCGGTCTGAA 3'
	Rev:5' TAGGGCTCGAGCGGCCGCCCGGGCAG 3'
Ribosomal protein S16	For: 5' GCTGTTGCTCACTGCAAACAGGGCAAAGGT 3'
	Rev: 5' TTGATCTCTTTCTTTGATGCTTCGTCGTC 3'
Nidogen secreted domain protein	For: 5' CAATGCAAGTATTGGCCATGGCATGGTAG 3'
	Rev: 5' ACCCAGCGTCCTGCTTTGGCGACGTTACT 3'
Lipid binding protein	For: 5' TTCAACATGTCTCAATTGAATGGGAAATGGAA 3'
	Rev: 5' TCCAGGCCCGTTTCGTTCACCTCGATACG 3'

## Competing interests

The authors declare that they have no competing interests.

## Authors' contributions

IB carried out gene amplification and library screening, the sequence analysis and drafted the manuscript. AT produced cDNA libraries and helped to draft the manuscript. DJ and BS sampled the experimental animals, designed and performed the experimental work on board of the ship N/O L'Atalante using the pressurized vessels IPOCAMP and helped to draft the manuscript. DM helped to draft the manuscript. All authors read and approved the final manuscript.
